# Determinants of fertility in rural Ethiopia: the case of Butajira Demographic Surveillance System (DSS)

**DOI:** 10.1186/1471-2458-11-782

**Published:** 2011-10-10

**Authors:** Wubegzier Mekonnen, Alemayehu Worku

**Affiliations:** 1 School of Public Health, College of Health Sciences, Addis Ababa University, Addis Ababa, Ethiopia

## Abstract

**Background:**

Fertility is high in rural Ethiopia. Women in the reproductive age group differed in various characteristics including access to food and encounter to drought which requisite the assessment of determinants of fertility.

**Methods:**

Reproductive age women were recruited from a DSS, the Butajira DSS database. A DHS maternity history questionnaire was administered on 9996 participants. Data quality was assured besides ethical clearance. Poisson regression crude and adjusted Incidence Rate Ratio with 95 Confidence Interval were used to identify determinants of fertility.

**Results:**

Delayed marriage, higher education, smaller family, absence of child death experience and living in food-secured households were associated with small number of children. Fertility was significantly higher among women with no child sex preference. However, migration status of women was not statistically significant.

**Conclusions:**

Policy makers should focus on hoisting women secondary school enrollment and age at first marriage. The community should also be made aware on the negative impact of fertility on household economy, environmental degradation and the country's socio-economic development at large.

## Background

The three components of population change (i.e., fertility, mortality and migration) have consequences on one another and the overall size and/or structure. In countries at the second stage of demographic transition, mortality reduction is followed by fertility decline [1]. However, drop in fertility is not yet witnessed in the general population of sub-Saharan Africa although it has been observed in some metropolitan areas and selected communities in the sub continent [2-4]. Ethiopia has never been unique in this regard as there were only half a child cut off between 2000 and 2005 from a total fertility rate of 5.9 to 5.4 children per woman [5,6]. Fertility is higher in rural compared to urban areas in the country. Nevertheless, below replacement level fertility of 1.9 children per woman was observed in Addis Ababa. The total fertility rate was however over 6 children per woman in rural Ethiopia. Moreover, there are regional disparities in fertility in the country. Southern Nations Nationalities and Peoples region (SNNPR), where this study was conducted, has one of the highest total fertility rates of 5.6 children per woman in 2005 [6].

Regardless, no detailed study on fertility that had policy implication in the context of current decentralization for such high fertility regions was recently done in Ethiopia. Various individual and household background characteristics of women influenced the level of fertility which required systematic assessment. The study area is also located in one of the densely populated and resource constrained parts of the country. Frequent food shortages, land degradation and population pressure lead residents to migrate and face high mortality of children under the age of five years [7-9]. This also gives an additional impetus to assess the effects of migration and childhood mortality on fertility in the study area. Therefore, the main purposes of this study are to assess determinants of fertility in rural Ethiopia, characterized by high child mortality and mobility resulting from population pressure, frequent episodes of drought and pestilence.

## Methods

This study was conducted in Butajira demographic surveillance system (DSS) started with 10 villages (9 rural and 1 urban) sampled according to probability proportional to size technique from 82 rural and 4 urban villages [10]. Butajira DSS is located in Guraghe zone of Southern Nations, Nationalities and People region (SNNPR) of Ethiopia. Residents of the study region varied in type of residential ecology, social, cultural, environmental, reproductive health and economic characteristics [11].

Active resident women in the reproductive age group recruited from the Butajira DSS database were interviewed during October-December 2009. The number of women in the reproductive age group living in the Butajira Demographic Surveillance Area (DSA) at the time of the survey was 11,133. A structured Demographic and Health Survey (DHS) type maternity history questionnaire was developed in English and translated into the local language of the respondents, and then back translated to English by an independent person. The questionnaire has been pilot tested in a different area prior to this study. Twenty clinical nurses and 5 supervisors all with a Bachelor degrees were recruited as data collectors and supervisors, respectively. Clinical nurses were recruited because advice on family planning use was part of the ethical consideration. Various data quality assurance mechanisms including using a standard data collection tool, recruitment of qualified female field staffs, intensive supervision, and mechanisms to minimize information contamination were put in place. Professional bias was over emphasized during the training prior to data collection to minimize it.

Data were entered into a template prepared on EPI INFO software with the CHECK program to manage internal consistency. Data were cleaned by reconciling inconsistencies. The cleaned data were exported to STATA version 11 for analysis. Total fertility rate (TFR), mean children ever born and Parity Progression Ratio (PPR) were computed. Moreover, Poisson regression [12,13] Incidence Rate Ratio (IRR) with 95 percent confidence interval (CI) was used to assess the association of various maternal and household characteristics with fertility. We checked that all assumptions of Poisson regression were fulfilled. Total children ever born to women in the reproductive age group which is a count data is considered as the outcome variable for this study. The overall significance of each covariate was first checked and those turned statistically significant were included in the bi-variate and multivariate Poisson regression model to compute crude and adjusted IRR. The reference category for each of the factors included in the model was selected based on a prior knowledge that women in this category had smaller fertility compared to the rest of the categories except for the case of household livelihood.

Ethical clearance was obtained from the Research and Ethics Committee of the School of Public Health; and Institutional Review Board of the College of Health Sciences of Addis Ababa University. Support letters were obtained from the districts, in which the study was conducted, through the Butajira DSS which hosted the study. Oral consent was also obtained from each study participant.

## Results

Though the overall size of women in reproductive age group in the Butajira DSA was 11133, we interviewed 9996 which resulted in a response rate of about 90%. The mean age of first marriage of study participants was estimated to be 16.9 years (SD = 2.4) with more than 80% of them married when they were aged between 15-19 years. Nearly half of the women had no formal education as shown in Table 1. Study participants were fairly distributed in different residential ecological zones. Having large household size appears to be an accepted norm as nearly 59 percent of study participants were living in households that had more than four family members. The average household size was around 5.2 persons (SD = 2.2). More than 65 percent of women in this study belonged to households whose main livelihood was farming. Sixty five percent of the study participants were born within surveillance villages. About 28 percent of the study participants lived in food-insecure households. About 43 percent of the interviewed women had incidence of child death. The majority (63.4%) of women knew when in the menstrual cycle pregnancy could occur if they had sex. Sixty two percent of women did not have sex preference for their children.

**Table 1:** The distribution of study participants by various characteristics in Butajira, 2009

Characteristics	Number	Percent
**Age at first Marriage**	6289	100.0
20 and over	838	13.3
15-19	5069	80.6
Less than 15	382	6.1
Mean (SD)	16.9 (2.4)	

**Educational status**	9996	100.0
Secondary plus	1172	11.7
Primary	3906	39.1
Never	4918	49.2

**Residential Ecology**	9996	100.0
Urban	3593	35.9
Rural Lowland	2999	30.0
Rural highland	3404	34.1

**Household size**	9995	100.0
Small (less or equal to 4)	4065	40.7
Large (greater than 4)	5930	59.3
Mean (SD)	5.2 (2.2)	

**Household livelihood**	9996	100.0
Farming	6503	65.1
Trade/Service	1606	16.1
Civil service	463	4.6
Other	1424	14.3

**Food shortage**	9994	100
No	7185	71.9
Yes	2809	28.1

**Migration status**	9994	100.0
In migrant	3496	35.0
Study site	6498	65.0

**Have a dead child**	5929	100.0
No	3358	56.6
Yes	2571	43.4

**Know pregnancy time**	9994	100.0
Yes	2548	63.6
No	1459	36.4

**Have child sex preference**	9994	100.0
Yes	3798	38.0
No	6196	62.0

**Figure 1:**
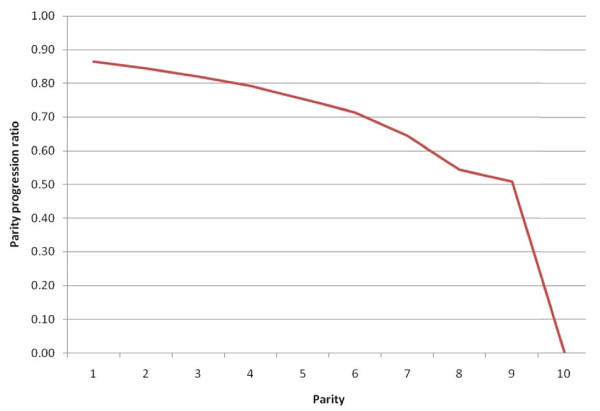
**Parity Progression Ratio for Women in Butajira, 2009**.

The mean children ever born to women in the reproductive age group was found to be 4.5 children (SD = 2.6) whereas the average number of children born to those in the age group (45-49 years) was 7.6 children. On the other hand, total fertility rate (TFR) was estimated to be 5.3 children and the total marital fertility rate was higher with 7.8 children per married woman. The age specific fertility rate revealed a typical developing country pattern (shown elsewhere). The parity progression ratio, the conditional probability of having the next parity given that the women had already a certain parity level, revealed that women of parity four had 79.1 percent chance of having the 5^th ^children (see Figure 1).

Analysis of determinants of fertility using Poisson regression Incidence Rate Ratio with 95 percent confidence interval showed that age at first marriage as a significant predictor of fertility even after including other variables in the model (Table 2). Fertility was 1.38 times higher among women married in their teens compared to those married after they celebrated their 20^th ^birthday. Educational status of women had also been consistently and significantly found to be negatively associated with fertility. Women who had never been into any formal education had 1.24 times more children compared to those who completed secondary and above level of education.

**Table 2:** Crude and Adjusted Incidence Risk Ratio of the Associations between maternal characteristics with Fertility in Butajira, 2009

Characteristics	Crude IRR (95%CI)	Adjusted IRR (95% CI)
Age at first Marriage		
20 and over	1.00	1.00
15-19	1.33 (1.27, 1.39)	1.11 (1.01, 1.20)*
Less than 15	1.83(1.75, 1.91)	1.38 (1.27, 1.49)*

Educational status		
Secondary plus	1.00	1.00
Primary	1.34 (1.24, 1.44)*	1.14 (1.02, 1.27)*
Never	1.83 (1.70, 1.97)*	1.24 (1.10, 1.39)*

Ecology		
Urban	1.00	1.00
Rural Lowland	1.29 (1.25, 1.33)*	0.88 (0.79, 0.98)*
Rural highland	1.33 (1.29, 1.38)*	0.92 (0.83, 1.03)

Household size		
Small (less or equal to 4)	1.00	1.00
Large (greater than 4)	2.08 (2.03, 2.15)*	1.95 (1.84, 2.06)*

Household livelihood		
Farming	1.00	1.00
Trade/Service	0.79 (0.77, 0.82)*	0.86 (0.78, 0.96)*
Civil service	0.66 (0.62, 0.71)*	0.96 (0.83, 1.11)
Other	0.67 (0.64, 0.71)*	0.90 (0.80, 1.01)

Food shortage		
No	1.00	1.00
Yes	1.16 (1.14, 1.19)*	1.06 (1.01, 1.13)*

Migration status		
In migrant	1.00	1.00
None-migrant	1.04 (1.02, 1.08)*	1.02 (0.97, 1.07)

Have a dead child		
No	1.00	1.00
Yes	1.90 (1.86, 1.95)*	1.67 (1.59, 1.76)*

Know correct pregnancy time		
Yes	1.00	1.00
No	1.35 (1,28, 1.41)*	1.09 (1.03, 1.15)*

Have sex preference		
Yes	1.00	1.00
No	1.14 (1.11, 1.17)*	1.09 (1.04, 1.15)*

*Statistically significant at α = 5 percent

Residential ecology composed of altitude and residence type in this study. The DSA comprised of lowland (below 1800 m), midland (188-2000 m) and highland (more than 2000 meters above sea level). Rural areas covered lowland and highland areas while Butajira fall in midland area. Residential ecology was significantly associated with fertility although the direction of association was changed when other covariates were included. Women resided in lowland rural Butajira had 1.3 times more children compared to those lived in the urban area. However, when other factors are included, women who lived in lowland rural Butajira had 12 percent lower fertility compared to urbanites. No fertility difference was observed between urban and highland rural Butajira when other factors were included. On the other hand, women who were members of a larger household (five plus) had about 2 times higher fertility compared to those who belonged to smaller households after other factors were added into the model.

Fertility among women whose households' main source of income was trade or service had 14 percent lower fertility compared to their counterparts whose household livelihood was farming after other factors were put into the model. On the other hand, women belonged to families whose household income was from the civil service had lower fertility compared to those earning their household income from farming although the statistical significance vanished when we control for other important variables. Meanwhile, the study was conducted in a drought prone area [7]. Women who were members of a food-insecure household had 6 percent higher fertility as compared to their counterparts in food secure households. The fertility of in-migrant women to the demographic surveillance area was lower than those who were born in the DSA although the association was statistically not significant when the effect of other vital variables were controlled. Women who had lost at least one of their children had about 1.7 times more fertility compared to their counterparts who never had an experience of child death. Fertility was about 9 percent higher in women who did not know the time at which women could be pregnant if they had sex. Similarly women who had no sex preference to their children had about 9 percent higher fertility compared to those with sex preference after including other significant covariates.

## Discussion

Total fertility and marital fertility rates of 5.3 and 7.8 (shown elsewhere) children per woman, respectively, obtained in this study was similar to the finding in Gondar, North West Ethiopia done in 2007 [14]. The fertility level is still one of the highest. This could be attributed to the credence of the wider community to large family size norm as children assisted households in subsistence farming and petty trade. Though disparities were observed across major regions of the world, children were considered as assets to their parents when they get older as shown in a study using the Demographic and Health Survey in 43 countries [15]. This posit was further supported by the statistically-significant finding of higher fertility among women who were members of larger households compared to those who belonged to smaller sized households (less or equal to 4 members) in this study. In this study, household constituted individuals regardless of their blood relations that live in one or more houses with the same cooking arrangement. Most members of the household were nuclear family members. The fact that women of parity 4 had more than 79 percent chance to have the 5^th ^parity augmented the deep rooted culture of larger family size that might have been supported in the study community in the foreseeable future. Thus, the total-fertility-rate goal stipulated in the Ethiopian population policy is far from reach [16].

Women that married in their teens had a significantly higher fertility compared to those married after they celebrated their 20^th ^birthday. Moreover, this study revealed that current contraceptive prevalence among women in the reproductive age group and married women were 15 and 25 percent, respectively (figure not shown). Besides women who marry early in life may have an increased risk of having many children, in particular, if they started childbirth before the age of 20 years. On the contrary, several studies [16,17] have shown that postponement of first childbirth to later ages leads to fertility reductions since women would have fewer years of reproduction window which may introduce parity specific controls even after the initiation of child birth.

On the other hand, women who had many years of education had significantly lower fertility as compared to those who had never been enrolled into any formal education system. This corroborates with similar studies [13,17-19] and may be attributable to the postponement of childbirth due to longer schooling. Educated women might be more worried to have many children if their area of usual residence had been stricken by frequent food shortage [7]. Nonetheless, a study among Sidamas in Southern Ethiopia indicated that fertility was higher among women with primary level of education compared to those who never attended any formal education. Higher educational level of women gives an opportunity of social and economic empowerments. Thus, able women might feel that they could take care of many children and opted for large family size. This is consistent with the claim by some researchers that increased family income leads to increased fertility when family planning use is low [17]. Education might also have impacts to bring about change in the knowledge and attitude towards low fertility. By the same token, women who did not know the time at which they could be pregnant had higher fertility as compared to those who knew it.

Disparities in level of fertility between urban and rural communities in this study population were similar to the finding in Gondar [18] that could be attributed to differences in contraceptive prevalence and age at first marriages between urbanites and rural residents in Ethiopia since women in urban areas had better access to media, general knowledge and services. It was however difficult to document reasons for the change in the direction of associations between residence ecology type and fertility when other factors were controlled. Meanwhile, informed urbanites might have a positive attitude towards smaller family size besides having better access to family planning services. For instance the urban fertility level of 3.3 children per woman found in this study (result not shown) was similar to the level found for Awassa town which is the capital of the study region [18]. In a similar context, fertility was significantly lower among women whose major livelihoods are based on trade or services compared to those households who got their main incomes from subsistence farming. Most of the households who relied on trade or services as main source of household income could be influenced by urban culture and practice which showed a low fertility norm.

Although food security was widely misunderstood, researchers considered it as shortages in the quality and quantity of food at any time while others defined it as shortage or absence of edible food [20]. In this study, encounter to household food scarcity in the past calendar year was collected. This variable might not precisely measure the household food security which could be cited as a limitation. However, it indirectly showed the household economic status and risk to vulnerability. Fertility was significantly higher among women whose households suffered from food shortages. There could be an egg-chicken dilemma in this claim since the food shortage might be caused by the large family size which in turn could mainly be triggered by higher fertility. In a resource constrained environment such as the study area, people had to share the scarce food and could probably be exposed to shortages particularly in rainy seasons, the time at which grain foods of farming households would be depleted [21]. Fertility was also found to be lower among malnourished mothers compared to nourished ones in a similar study done for Sidamas in Southern Ethiopia [22].

An in-migrant to the DSA is a person who went there to live or stayed in it for 6 or more months if did not have such intention. No significant association has been shown between migration status of women and fertility in this study. This could be due to the fact that the duration of residence of in-migrants may not be long enough to influence fertility and reproductive health behavior in the study area as migration was categorized broadly. It may also that in-migrant women could have come from rural villages in the same or nearby districts of the country which had similar socio-cultural and behavioral characteristics. Nevertheless, a study in China had indicated that rural-urban return migrants from middle and large cities had adapted positive fertility behavior towards small family size norm compared to their rural-rural return migrant counterparts [23]. The same study had also revealed that areas with higher prevalence of rural-urban return migrants particularly from middle and mega cities would have a positive effect to adapt small family size.

Studies done on fertility responses to childhood mortality have focused on insurance and replacement effects [24]. Couples in high-mortality settings anticipate the death of some of their children that might dictate them to change their reproductive preferences and behavior. Childhood mortality could also have the combined biological and volitional replacement effects to reduce the time to subsequent conception if the death occurs within a given interval. The time to conception could also be reduced if a childhood death occurs during a prior birth interval. Accordingly, this study had revealed child mortality as a strong and significant predictor of fertility as documented for the same study population a decade ago and elsewhere [17-19]. Epidemic and frequent episodes of drought in the study community, which might have claimed the lives of their children, provoked mothers to replace the lost ones [7].

Contrary to the findings of many studies, fertility was significantly higher among women who had no preference to the sex of their children. Conversely, studies done in Ethiopia and Asia had revealed more preference towards sons compared to daughters because males inherit properties from their ancestors in a patrilineal society. A similar study in USA indicated a stable marital union or request for the custody of their child by fathers if the marriage was dissolved when couples had sons instead of daughters [22,25,26]. Religiosity among women in the study community had probably dimmed their decision.

The use of Butajira DSS database to recruit study participants and administration of standard maternity history questionnaire by clinical nurses could be mentioned as strengths for the current study. The demographic surveillance staffs knew study participants for a long time and this built trust on the participants side to provide reliable information. The study was also conducted in a peak harvesting season which reduced the participation of certain community groups that could be away from their home by virtue of their work.

## Conclusions

Local government authorities should play pivotal role to make women stay more years at school thereby increase the age at first marriage to reduce fertility in the area. Special attention should also be given to increase the enrollment of women in secondary education to significantly reduce fertility in rural communities. Moreover, the community should be made aware on the negative impact of large family size on the household economy, environmental degradation and the country's socio-economic development at large. Efforts to enhance child survival have to be scaled up to curve the level of fertility in resource-constrained rural Ethiopia.

## Competing interests

The authors declare that they have no competing interests.

## Authors' contributions

WM participated from conception to the final approval of the final version of the article. AW supervised the whole exercise and made critical comments at each step in the research. He also approved the final version of the article.

## Pre-publication history

The pre-publication history for this paper can be accessed here:

http://www.biomedcentral.com/1471-2458/11/782/prepub

## References

[B1] Caldwell JC Toward A Restatement of Demographic Transition Theory Population and Development Review 1976 2 3/4 321 66 10.2307/1971615

[B2] Caldwell JC Caldwell P High fertility in sub-Saharan Africa Sci Am 1990 262 5 118 25 10.1038/scientificamerican0590-118 2333491

[B3] Sibanda A Woubalem Z Hogan DP Lindstrom DP The proximate determinants of the decline to below-replacement fertility in Addis Ababa, Ethiopia Stud Fam Plann 2003 34 1 1 7 10.1111/j.1728-4465.2003.00001.x 12772441

[B4] Gurmu E Mace R Fertility decline driven by poverty: the case of Addis Ababa, Ethiopia J Biosoc Sci 2008 40 3 339 58 18190731 10.1017/S002193200700260X

[B5] CSA, ORC-Macro Ethiopia Demographic and Health Survey 2000: Central Statistical Authority, Addis Ababa, Ethiopia; ORC Macro, Calverton, USA 2001

[B6] CSA, ORC-Macro Ethiopia Demographic and Health Survey 2005: Central Statistical Authority, Addis Ababa, Ethiopia; ORC Macro, Calverton, USA 2006

[B7] Emmelin A Fantahun M Berhane Y Wall S Byass P Vulnerability to episodes of extreme weather: Butajira, Ethiopia, 1998-1999 Glob Health Action 2009 2 10.3402/gha.v2i0.1829 PMC2799308 20052373

[B8] Byass P Fantahun M Emmelin A Molla M Berhane Y Spatio-temporal clustering of mortality in Butajira HDSS, Ethiopia, from 1987 to 2008 Glob Health Action 3 10.3402/gha.v3i0.5244 PMC2935921 20838630

[B9] Grepperud S Population pressure and land degradation: the case of Ethiopia J Environ Econ Manage 1996 30 1 18 33 10.1006/jeem.1996.0002 12292334

[B10] Shamebo D Sandstrom A Wall S The Butajira rural health project in Ethiopia: epidemiological surveillance for research and intervention in primary health care Scand J Prim Health Care 1992 10 3 198 205 10.3109/02813439209014061 1410950

[B11] CSA Summary and Statistical Report of the 2007 Population and Housing Census Results of Ethiopia 2008 Addis Ababa: Federal Democratic Republic of Ethiopia Population Census Commission

[B12] StataCorp STATA Version 11. 11 ed. College Station, Texas 77845 USA 2009

[B13] Bhargava A Desired family size, family planning and fertility in Ethiopia J Biosoc Sci 2007 39 3 367 81 10.1017/S0021932006001593 16928286

[B14] Alene GD Worku A Estimation of the total fertility rates and proximate determinants of fertility in North and South Gondar zones, Northwest Ethiopia: An application of the Bongaarts' model EthiopJHealth Dev 2009 23 1 19 27

[B15] Bongaarts J Zimmer Z Living Arrangements of Older Adults in the Developing World: An Analysis of Demographic and Health Survey Household Surveys Journal of Gerontology: Social Sciences 2002 57 3 S145 S57 10.1093/geronb/57.3.s145 11983741

[B16] TGE The National Population Policy of Ethiopia 1993 Addis Ababa, Ethiopia

[B17] Alene GD Worku A Differentials of fertility in North and South Gondar zones, northwest Ethiopia: a comparative cross-sectional study BMC Public Health 2008 8 397 10.1186/1471-2458-8-397 19055705 PMC2610031

[B18] Gebremedhin S Betre M Level and differentials of fertility in Awassa town, Southern Ethiopia Afr J Reprod Health 2009 13 1 93 112 20687268

[B19] Fitaw Y Berhane Y Worku A Impact of child mortality and fertility preferences on fertility status in rural Ethiopia East Afr Med J 2004 81 6 300 6 16167677 10.4314/eamj.v81i6.9179

[B20] Smith M Pointing J Maxwell S Sussex I Household Food Security: Concepts and Definitions - An Annotated Bibiliography

[B21] Kaluski DN Ophir E Amede T Food security and nutrition - the Ethiopian case for action Public health Nutrition 2001 5 3 373 81 10.1079/phn2001313 12003647

[B22] Regassa N Socio-economic correlates of High Fertility among Low Contraceptive Communities of Southern Ethiopia JHumEcol 2007 21 3 203 13

[B23] Chen J Liu H Xie Z Effects of Rural-Urban Return Migration on Women's Family Planning and Reproductive Health Attitudes and Behavior in Rural China Stuides in Family Planning 2010 41 1 31 44 10.1111/j.1728-4465.2010.00222.x 21465720

[B24] Hossain MB Phillips JF Legrand TK The impact of childhood mortality on fertility in six rural thanas of Bangladesh Demography 2007 44 4 771 84 10.1353/dem.2007.0047 18232210

[B25] Pong S-l Sex Preference and Fertility in Peninsular Malaysia Stuides in Family Planning 1994 25 3 137 48 10.2307/2137940 7940619

[B26] Raley S Bianchi S Sons, Daughters and Family Process: Does Gender of Children Matter? Annu Rev Sociol 2006 32 401 21 10.1146/annurev.soc.32.061604.123106

